# Single-cell Transcriptome Study as Big Data

**DOI:** 10.1016/j.gpb.2016.01.005

**Published:** 2016-02-11

**Authors:** Pingjian Yu, Wei Lin

**Affiliations:** Genomics and Bioinformatics Lab, Baylor Institute for Immunology Research, Dallas, TX 75204, USA

**Keywords:** Single cell, RNA-seq, Big data, Transcriptional heterogeneity, Signal normalization

## Abstract

The rapid growth of **single-cell RNA-seq** studies (scRNA-seq) demands efficient data storage, processing, and analysis. **Big-data** technology provides a framework that facilitates the comprehensive discovery of biological signals from inter-institutional scRNA-seq datasets. The strategies to solve the stochastic and heterogeneous **single-cell** transcriptome signal are discussed in this article. After extensively reviewing the available **big-data** applications of next-generation sequencing (NGS)-based studies, we propose a workflow that accounts for the unique characteristics of scRNA-seq data and primary objectives of **single-cell** studies.

## Introduction

Multi-institutional collaborative omics studies on the next-generation sequencing (NGS) platform have generated petabytes of data that constitute ‘big data’ from the perspective of scale and complexity [1], [2], [3], [4], [5], [6]. Particularly, transcriptomics studies using the RNA-seq technique have become revolutionary and powerful [7], [8], [9]. Scientists have now moved one step forward to single-cell RNA sequencing (scRNA-seq) by employing new protocols for single cell isolation, low-input RNA extraction, reverse transcription, and unbiased amplification [9], [10], [11], [12], [13]. Given the high anticipated value of single-cell transcriptomics, explosive growth of scRNA-seq data is expected in the next 5–10 years. Consequently, uncovering the hidden pattern, connectivity, and interactions of such huge and heterogeneous data will be a major challenge.

Without a doubt, the detailed and extremely-valuable information that single-cell technology provides is at a significant cost due to sophisticated data acquisition, large data-storage requirements, as well as challenging data processing and management. Big data incorporate a body of technologies including computational parallelization and distribution, data visualization, and data integration that are used to reveal the hidden associations within large datasets that are diverse, complex, and of a massive scale. Data-intensive scientific discovery has been proposed as the 4th paradigm of scientific research [14], following and interacting with the other three paradigms – theory, experimentation, and simulation modeling. In 2001, Doug Laney defined characteristics of big data in three dimensions, *i.e.*, increasing volume (amount of data), velocity (speed of data I/O), and variety (range of data types and sources) [15]. While agreeing that volume, variety, and velocity are the quantitative characteristics of big data, Ivanov et al. [16] added that variability (the inconsistency the data can show over time) and veracity (the quality of captured data) are the qualitative characteristics of big data.

**Figure 1:**
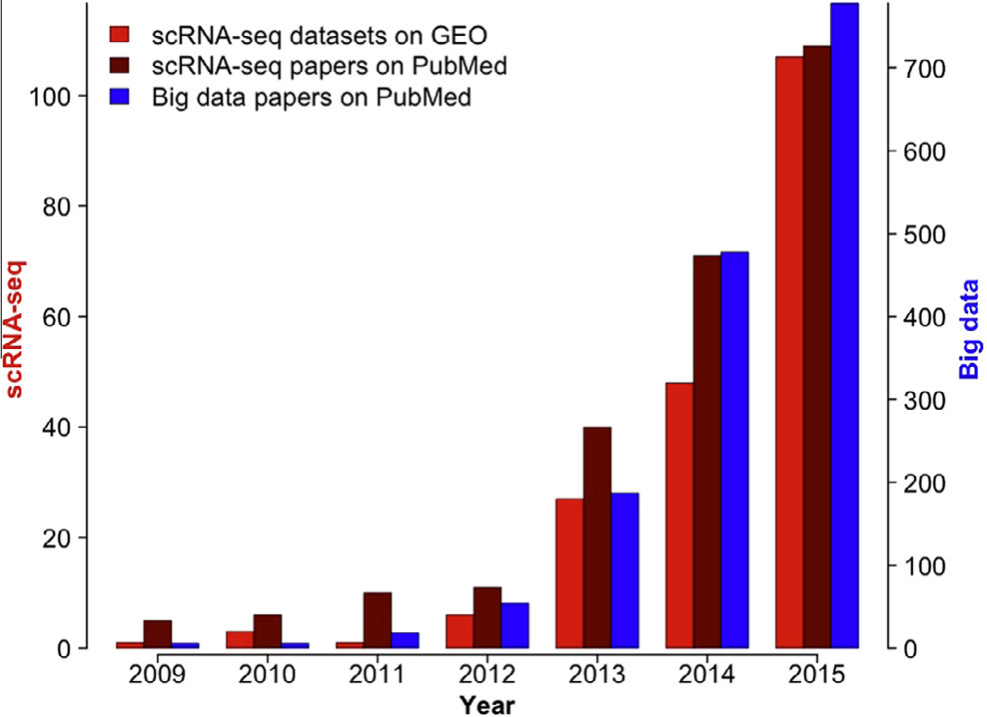
**Number of papers/datasets addressing single-cell data and big data** Searches were performed on January 04, 2016 on http://www.ncbi.nlm.nih.gov/gds for datasets and http://www.ncbi.nlm.nih.gov/pubmed for papers. Data were obtained according to the search criteria as follows filtered by year: (1) for scRNA-seq datasets on GEO: “single cell”[All Fields] AND “Expression profiling by high throughput sequencing”[Filter]; (2) for scRNA-seq papers on PubMed: “single cell”[All Fields] AND (“rna-seq”[All Fields] OR “rna sequencing”[All Fields] OR (“sequencing”[All Fields] AND “transcriptome”[All Fields])); and (3) for big-data papers on PubMed: “big data”[All Fields] OR “hadoop”[All Fields].

Big-data technology has many applications in biomedical research [17], [18], [19], [20]. Particularly, high-throughput molecular and functional profiling of patients using NGS or single-cell technology is the key driving force of precision medicine [21], [22], [23], [24]. By examining the annual growth of scRNA-seq datasets uploaded to the NCBI Gene Expression Omnibus (GEO) database [25] and the increasing number of new articles in PubMed over the past 7 years that involve scRNA-seq and big-data (Figure 1), we expect the extensive integration of big data and scRNA-seq technologies.

In the following sections, we will discuss the characteristics of single-cell transcriptomics, especially scRNA-seq, data as examples of big data. We will discuss how to adapt single-cell transcriptomics study to big-data infrastructure such as Hadoop and MapReduce.

## Transcriptional stochasticity and cellular heterogeneity

**Figure 2:**
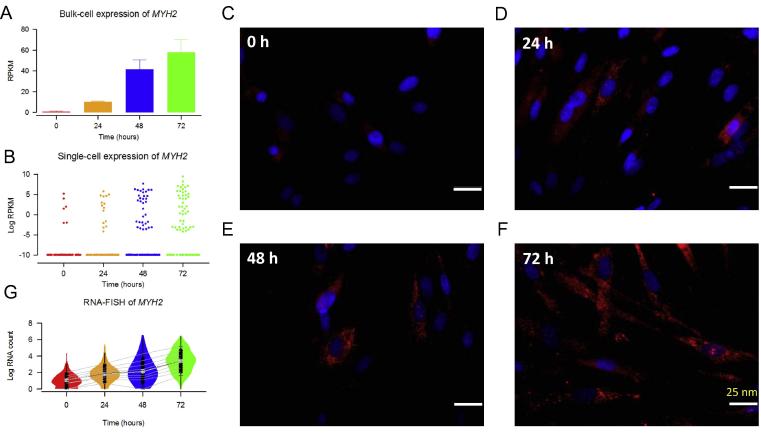
***MYH2* gene is the marker of mature myotubes** The increased bulk expression of *MYH2* is primarily driven by the growing proportion of “on-” component cells (upper cluster) over time (0, 24, 48, and 72 h after myoblast differentiation is induced). Figures were derived from the dataset in Trapnell et al [41]. **A.** The growth of *MYH2* expression in bulk cell replicate samples (*n* = 3 over time). **B.** Beeswarm plots of the growing bimodal proportion of *MYH2* from scRNA-seq over time. **C**–**F.** RNA-FISH signals at 0, 24, 48, and 72 h, respectively. *MYH2* and nucleus are shown in red and blue (DAPI staining), respectively. Scare bar: 25 nm. **G**. *MYH2* RNA molecule counts per cell over time, based on RNA-FISH analyses. RNA-FISH, RNA-fluorescence *in situ* hybridization.

scRNA-seq is always compared to bulk RNA-seq in terms of signal profile and noise level. In addition to the descriptive keyword like high resolution, stochasticity and heterogeneity are also frequently used to feature the single-cell transcription [26], [27], [28], [29]. Most of the scRNA-seq investigators have experience with zero-inflation transcriptional signals. Some of them tend to regard this phenomenon as technical dropout. We prefer to use the phrase “bimodality” to delineate the signal distribution, since recent results have shown that the low transcriptional values are biologically meaningful signals rather than technical dropout. Shalek et al. have revealed the bimodality of single-cell expression and splicing using both scRNA-seq and RNA fluorescence *in situ* hybridization (RNA-FISH) [30]. The two modes in an expression profile can be attributed to the “on” or “off” transcriptional status. Figure 2 demonstrates two clusters of cells showing different expression level and the change of the ratio of on/off status of a marker gene *MYH2* over time during human myoblast cell differentiation using both scRNA-seq and RNA-FISH [31]. The aforementioned studies indicate that even from a seemingly homogeneous population, many genes are expressed in a stochastically-bursting fashion and their abundance exhibits a bimodal distribution in the cell population examined. The traditional RNA-seq analysis method rarely takes such transcriptional bimodality into account. Further investigation on co-bursting networks have validated the biological significance of the “bimodality” rather than just relegating it to technical dropout [31].

Several computational models have been proposed to analyze transcriptional stochasticity and cellular heterogeneity in scRNA-seq data in the context of zero-inflation or bimodality. Kim and Marioni [32] use a mixture of two Poisson distributions to model theoretical kinetics for ‘bursty’ gene expression. However, in the presence of massive variability, the model is compromised by excessive over-dispersion in read counts. Kharchenko et al. take the probability of “dropout” into consideration in their differential-expression algorithm [33]. Pierson and Yau proposed using zero-inflated factor analysis to perform dimensionality reduction [29]. Gu et al. use a mixture of two negative binomial distributions to model over-dispersed read counts generated from a gene’s two distinct biological states: an ‘on’ component and an ‘off’ component [31]. All of these four studies acknowledge the fact that single-cell transcription signals cannot be solved by unimodal statistics. Gu et al. first introduced the statistics term “bimodal proportion” to measure the ratio of two signal modes in a single-cell population. The functional enrichment of co-bursting transcription supports the biological significance of transcriptional bursting over technical dropout. The value of “bimodal proportion” ranges from 0 to 1 and notably, it can be compared across different datasets without additional normalization.

## The opportunities and challenges of scRNA-seq

Single-cell transcriptomics provides us unprecedented opportunity to understand the transcriptional stochasticity and cellular heterogeneity in great detail, which are crucial for maintaining cell functions and for facilitating disease progression or treatment response [34], [35], [36], [37], [38]. Such stochasticity and heterogeneity are always masked in bulk-cell studies [27]. Recent single-cell applications have utilized a broad range of tissues [28], [39], [40], [41], [42], stem cell lines [43], [44] and cell populations with clinical backgrounds [45]. The cell types that have been interrogated using scRNA-seq in the GEO database are briefly summarized in Table 1.

scRNA-seq is one of the most promising technologies for single-cell transcriptomics [46], [47]. Nevertheless, it also poses big challenges, largely stemming from the aforementioned big-data characteristics with regard to the data management, query, and analysis. There are five ‘V’s to consider for scRNA-seq data. (1) Volume. NGS data has become one of the largest big-data domains in terms of data acquisition, storage, and distribution [48]. Just like bulk-cell RNA-seq and other NGS-based studies, scRNA-seq generates a high volume of raw sequencing data and high-dimensional transformed expression data. Moreover, due to the heterogeneity of cell populations, a typical scRNA-seq study usually incorporates hundreds or even thousands of cells and thus adds a few more orders of magnitude to the data volume. (2) Velocity. As aforementioned, the data volume of scRNA-seq is higher than that of bulk-cell RNA-seq. Consequently, high data-transfer bandwidth, parallel algorithms, and high-performance computers are required to generate and process data. (3) Variety. An scRNA-seq study may combine data from different single-cell isolation chips, protocols, and research environments. How to normalize the datasets and make them comparable becomes a major issue. (4) Variability. The transcriptional activity of a living cell is dynamic rather than static. Thus, scRNA-seq captures a snapshot of single cells in seemingly homogeneous populations that as a matter of fact, vary significantly from one to another. Substantial variability of the scRNA-seq signal comes from a variety of biological aspects, including transcriptional stochasticity and cellular heterogeneity, which cannot be investigated in bulk-cell studies. Therefore, scRNA-seq data exhibit significantly larger variance than bulk-cell RNA-seq data [33]. Solving the biological variability is the main goal of single-cell transcriptomics research. (5) Veracity. scRNA-seq is composed of sequential steps of target cell isolation, RNA extraction, fragmentation, reverse transcription, cDNA amplification, sequencing, alignment, and read counting. Every step introduces biases and artifacts that may significantly affect the coverage, accuracy, and timeliness of transcript expression and thus interfere with both the proper characterization and quantification of transcripts. It is therefore critical to control the data quality prior to including the datasets in a meaningful global study.

Due to the much lower starting amount of RNA in a single cell, it takes more cycles of amplification using a template-switching strategy, compared to the bulk-cell sequencing [49], [50] and thus introduces much larger technical variations to the scRNA-seq data. Technical variations in scRNA-seq include but not limit to the ones introduced by RNA extraction, transcript fragmentation, reverse transcription, PCR amplification, sequencing sampling, sequencing error, short-read mapping error, and miscount. Because the technical variation introduced during earlier steps will be carried over to the later steps and even be amplified further, it is critical to control the technical variations in the earlier steps. Artificial RNA molecules such as the External RNA Controls Consortium spike-in molecules (ERCC) can be doped into the assayed RNA samples at the same level. Since there is no expected biological variation for the ERCC transcripts in the samples, the variation in the ERCC quantification measurements in the scRNA-seq will be due to technical variability. This is a reliable way to quantify technical variation in scRNA-seq [51]. Technical variations may confound with biological variations, and we can only observe total variation in gene expression. Efforts have been made to distinguish the technical variation from biological variation in scRNA-seq by computational methods with or without ERCC control [52], [53].

The best efforts at mitigating the technical variations have been made by protocol modification. Saliba et al. [10] and Kolodziejczyk et al. [54] have reviewed a variety of single-cell RNA-seq techniques. Besides including external molecule controls, improved single-cell chemistry and physics [8], [9], [55], as well as incorporation of molecular barcoding system [56], have significantly reduced the noise level within each study.

## Big data—the norm of NGS technology

A typical RNA-seq study on the most popular NGS platform such as the Illumina HiSeq 2500 usually generates hundreds of gigabytes (GB) of raw read data. It usually takes hours to align these raw reads to the human or other mammalian reference genomes. The NGS throughput and computer processors are in a race and the growth of NGS data always seems to win [57]. Moreover, a robust data storage, management and analysis framework is in need.

The National Center for Biotechnology Information (NCBI) hosts RNA-seq data using two data storage/sharing platforms, *i.e.*, Gene Expression Omnibus database (GEO) [25], [58] and Sequence Read Archive database (SRA) [59]. Both of these databases provide comprehensive metadata structure, including information about the data producer, study design, sample description, technical details, keywords, *etc*. The metadata that they collect has been considered as data-sharing standards and the overall bioinformatics infrastructure in a big-data system.

Apache Hadoop is an open-source software framework for distributed storage and distributed processing of very large datasets on computer clusters. The key modules in the Apache Hadoop framework are the Hadoop Distributed File System (HDFS) and Hadoop MapReduce. Apache Hadoop uses its HDFS to store data on commodity machines, providing very high aggregate bandwidth across the cluster. In addition, Apache Hadoop implements MapReduce technology [60] to decompose a large-scale problem into small independent sub-problems and schedule the sub-problems to computer clusters. MapReduce allows the development of approaches that can handle larger volumes of data using a larger number of processors simultaneously. By utilizing parallel-based approaches, Apache Hadoop improves the flexibility and scalability of computer clusters.

scRNA-seq utilizes the most common short-read mapping, as well as data storage and query procedures of common NGS applications. The Hadoop-based bioinformatics applications [61], [62], [63], [64], [65], [66], [67], [68], [69], [70], [71], [72], [73], [74], [75], [76], [77], [78], [79], [80], [81], [82], [83], [84], [85], [86], [87], [88], [89], [90], [91] are reviewed in Table 2. To our best knowledge, there is no Hadoop application specially designed for scRNA-seq so far. Given the unique bimodal signal profile of scRNA-seq data, the long-used unimodal statistics in bulk RNA-seq cannot satisfy the need to determine differential expression in scRNA-seq. It has also been validated that change of bimodal proportion/burst frequency as well as the coordination of transcriptional bursts are biologically meaningful [31]. Thus the new analytic components should be included when mining the scRNA-seq data in the big-data domain.

A robust normalization underlies the success of analyses across datasets. The goal of using a big-data approach for scRNA-seq studies is not just to take full advantage of the computational resources on the cloud but also to integrate the sample power of multiple single-cell datasets to uncover the global associations and the molecular mechanisms that maintain the cellular function of biological systems. Reference like ERCC for signal normalization, as discussed above, can be added on the bench side. On the computational side, signal-rescaling algorithms (Table 3) based on the putative abundance of internal references (*e.g.*, ERCC and housekeeping genes) can be implemented. Reads per million mapped reads (RPM), reads per kilobase per million mapped reads (RPKM) [92], median, and upper-quantile normalizations [93] rescale the raw counts based on mean, mean with gene length considered, median, and upper-quantile of read counts, respectively, in a sample. Full-quantile normalization [94] aligns all quantiles of the count distributions among samples. Other than direct comparison of the rescaled RNA abundance signal across samples, dataset normalization also involves a variety of analyses, including statistical modeling and hypothesis testing that are used to delineate and compare the read-count-based profiles of samples and datasets (Table 3). GC-content [95], DESeq [96], trimmed mean of M values (TMM) [97], remove unwanted variation (RUV) [98], Poisson beta [32], and Sphinx [31] utilize statistical modeling to infer normalized read counts. Owing to the distribution assumed, DESeq, TMM, RUV, Poisson beta, and Sphinx allow over-dispersion on read counts. In particular, Poisson beta and Sphinx can identify transcriptional status through bimodality, which characterizes the single-cell RNA-seq signal profiles. Because of the regression method used, GC-content, DESeq, TMM, and RUV can model batch effect and other known factors such as cycles of PCR amplification and length distribution of fragments.

**Figure 3:**
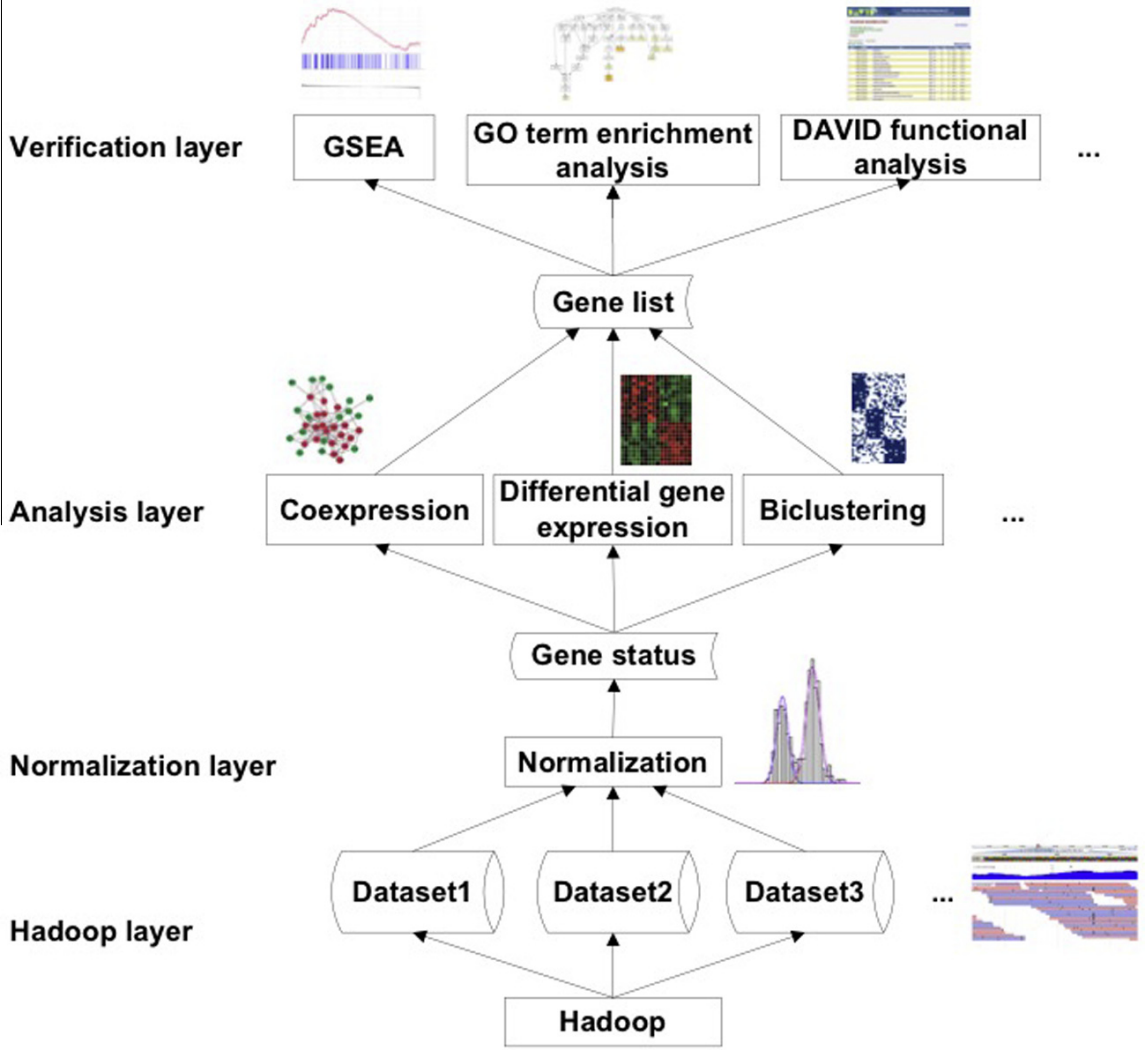
**Workflow of inter-institutional scRNA-seq data integration** Inter-institutional single-cell RNA-seq datasets are aligned against their genomes at the Hadoop layer. Read counts are resolved into gene “on” or “off” status at the normalization layer. Differential expression, co-expression, and other applications are developed based on gene “on” or “off” status instead of gene expression. Biology in the resulting gene list is verified by GSEA, GO-term enrichment analysis, DAVID functional analysis or other tools. GSEA, gene set enrichment analysis; GO, gene ontology; DAVID, database for annotation, visualization and integrated discovery.

We hereby propose a workflow for inter-institutional scRNA-seq data integration and analysis (Figure 3). The workflow consists of four layers: Hadoop, normalization, analysis, and verification. (1) Hadoop layer. Inter-institutional scRNA-seq data is stored and managed in this layer using HDFS. Parallel algorithms, such as short-read alignment and read count per transcript algorithms, can be implemented under Hadoop framework provided in this layer. (2) Normalization layer. To make the single-cell expression profiles comparable across different studies or even across different chips/runs for the same study, normalization is not just the most important task but also the biggest challenge. Normalization covers the analyses that are performed to control the cross-assay technical variation. Nonetheless, different normalization strategies should be extensively tested and compared. As discussed, single-cell RNA-seq exhibits unique bimodal transcriptional profiles that can be resolved into “on” and “off” components. This unique transcriptional pattern distinguishes the single-cell RNA-seq analyses from traditional bulk-cell RNA-seq and provides a naturally normalized signal profile for comparison. (3) Analysis layer. In this layer, the normalized single-cell gene expression profiles are loaded as input. The output of the analysis is the target gene sets that drive the divergence of the cellular phenotypes or experimentally-controlled cellular groups. Determination of differential expression and co-expression, as well as biclustering will be implemented in this layer to identify the pattern in gene expression profiles. The target gene sets or the classification of the cell populations will be further interpreted in the verification layer. (4) Verification layer. In this layer, the biological significance of the input gene set will be analyzed, interpreted, and verified using tools such as the gene set enrichment analysis (GSEA), and gene ontology (GO)-term enrichment analysis, as well as the database for annotation, visualization and integrated discovery (DAVID) functional analysis, *etc*.

## Outlook

Big data and scRNAseq are two rapid-growing technologies. Big data not only can provide the framework to host, process, transform, and visualize the data from different sources, but also can increase the sample power by including comprehensive sample descriptions and ruling out cross-study batch effects. Notably, the big-data framework offers the opportunity to identify significant correlation in new dimensionalities, with the sample power that cannot be reached by individual studies on these dimensionalities. One possible application of big data for scRNA-seq is in mammalian single-cell studies, which are often associated with the origin of cells from different body parts. This means the assayed single cells can be mapped spatially. The atlas of cell phenotypes or interactive behaviors can be further explored in this way. This spatial data infrastructure has been widely used in geoinformatics and has now become a popular methodology of big data. For instance, the Human Protein Atlas project is one of the research efforts that is taking the idea to the protein level [99]. As the vehicles of the DNA, RNA, and protein molecules, single cells carry the molecular signature of the phenotypic and functional elements. They should also be able to be systematically assayed and organized in the big-data domain.

Many diseases, especially cancer, are heterogeneous when considered from two different perspectives. On the one hand, cancer tissues are heterogeneous and thus require the high-resolution information that can be obtained from single-cell technology. On the other hand, certain cancer categories are actually defined from a heterogeneous patient population that requires personalized solutions. Big-data technology has been recognized by Doudican et al*.* for its ability to inform personalized therapeutics [100]. Irish and Doxie have recently reviewed the progress of applying single-cell technology to cancer biology [101], and the advancements are significant. The big-data infrastructure of the ever-increasing number of single-cell RNA-seq datasets will eventually facilitate the decisions that are based on the comparison of clinical sample characteristics at a higher resolution, as well as interrogation of previous treatment responses within larger datasets.

## Competing interests

The authors have declared no competing interests.

## Figures and Tables

**Table 1:** Summary of cell types in GEO datasets

**Cell type**	**No. of datasets**
Neuron	11
Embryonic	80
Blood	18
Lung	17
Renal	4
Brain	17
Skin	26
Heart	9
Bone marrow	17
Stem cell	43
Tumor	23
Cell line	71

Total No. of unique datasets	195

**Table 2:** Hadoop-based bioinformatics software tools

**Function**	**Name**	**Weblink**	**Description**	**Ref.**
Sequence file management	LFQC	http://engr.uconn.edu/rajasek/lfqc-v1.1.zip	A lossless compression algorithm for FASTQ files	[61]
Quake	http://www.cbcb.umd.edu/software/quake	Quality-guided error detection and correction of short reads	[62]
SeqPig	http://sourceforge.net/projects/seqpig/	Simple and scalable scripting for large sequencing datasets	[63]
Hadoop-BAM	http://sourceforge.net/projects/hadoop-bam/	Library for scalable manipulation of aligned NGS data	[64]
smallWig	http://publish.illinois.edu/milenkovic/	Parallel compression of RNA-seq WIG files	[65]

Search engine	SeqWare	http://seqware.sourceforge.net	Pipeline and query engine for storing and searching sequence	[66]
Hydra	http://code.google.com/p/hydra-proteomics/	A protein sequence database search engine	[67]
SparkSeq	https://bitbucket.org/mwiewiorka/sparkseq/	Interactive data querying of genomic data analysis	[68]
GMQL	http://www.bioinformatics.deib.polimi.it/GMQL/	Large-scale genomic data query and management	[69]

Genomic sequence mapping	CloudAligner	http://mine.cs.wayne.edu:8080/CloudAligner/	A MapReduce-based application for short read alignment	[70]
CouldBurst	http://cloudburst-bio.sourceforge.net/	A parallel short read mapper	[71]
BigBWA	https://github.com/citiususc/BigBWA	Hadoop implementation of BWA	[72]
SEAL	http://biodoop-seal.sourceforge.net/	Alignment, manipulation, and analysis of short reads	[73]
DistMap	http://code.google.com/p/distmap/	A toolkit for distributed short read mapping	[74]
SOAP3	http://www.cs.hku.hk/2bwt-tools/soap3	Short sequence read alignment with GPU acceleration	[75]
GPU-BLAST	http://archimedes.cheme.cmu.edu/biosoftware.html	NCBI-BLAST with GPU acceleration	[76]

Expression analysis	Myrna	http://bowtie-bio.sf.net/myrna	RNA sequencing differential expression analysis	[77]
Eoulsan	http://transcriptome.ens.fr/eoulsan/	Pipeline for calculating differential gene expression	[78]
YunBe	http://tinyurl.com/yunbedownload	A gene set analysis algorithm for biomarker identification	[79]
FX	http://fx.gmi.ac.kr	Gene expression estimation and genomic variant calling	[80]

Phylogenetic analysis	FVGWAS	http://www.nitrc.org/projects/fvgwas	Fast voxel-wise genome-wide association analysis	[81]
GATK	http://www.broadinstitute.org/gsa/wiki/index.php/The_Genome_Analysis_Toolkit	Variant calling	[82]
Crossbow	http://bowtie-bio.sourceforge.net/crossbow/	Alignment and SNP genotyping with Bowtie and SoapSNP	[83]
MrsRF	http://mrsrf.googlecode.com	Calculate Robinson–Foulds distance between trees	[84]
BlueSNP	http://github.com/ibm-bioinformatics/bluesnp	Genome-wide association studies using Hadoop clusters	[85]
GeneCOST	www.igbam.bilgem.tubitak.gov.tr/en/softwares/genecost-en/index.html	Scoring-based prioritization to identify disease-causing genes	[86]
Nephele	http://code.google.com/p/nephele/	Genotyping via complete composition vector	[87]

Miscellaneous	PeakRanger	http://www.modencode.org/software/ranger/	A cloud-enabled peak caller for ChIP-seq data	[88]
SeqHBase	http://seqhbase.omicspace.org	A big-data toolset for family-based sequencing data analysis	[89]
ProKinO	http://vulcan.cs.uga.edu/prokino	A unified resource for mining the cancer kinome	[90]
BioPig	https://sites.google.com/a/lbl.gov/biopig/	An analytic toolkit for large-scale sequence data	[91]

**Table 3:** Read count normalization methods

**Name**	**Normalization method**	**Assumed distribution**	**Parameter estimation**	**Over-dispersion capability**	**Gene status identification capability**	**Correction factor**			
**Sequencing depth**	**Gene length**	**GC content**	**Batch**
RPM	Rescale	N/A	N/A	No	No	Yes	No	No	No
RPKM	Rescale	N/A	N/A	No	No	Yes	Yes	No	No
Median	Rescale	N/A	N/A	No	No	Yes	No	No	No
Upper-quantile	Rescale	N/A	N/A	No	No	Yes	No	No	No
Full-quantile	Rank average	N/A	N/A	No	No	Yes	No	No	No
GC-content	Statistical model	Non-parametric	Local regression	No	No	Yes	Yes	Yes	Yes
DESeq	Statistical model	Negative binomial	GLM	Yes	No	Yes	No	No	Yes
TMM	Statistical model	Negative binomial	GLM	Yes	No	Yes	No	No	Yes
RUV	Statistical model	Lognormal	GLM	Yes	No	Yes	No	No	Yes
Poisson beta	Statistical model	Mixed Poisson	Bayesian	No	Yes	Yes	No	No	No
Sphinx	Statistical model	Mixed negative binomial	Bayesian	Yes	Yes	Yes	No	No	No

*Note:* RPM, reads per million mapped reads; RPKM, reads per kilobase per million mapped reads; TMM, trimmed mean of M values; RUV, remove unwanted variation; GLM, generalized linear model.
